# The mediating effect of regulatory emotional self-efficacy between work-family conflict and turnover intention in female nurses with two children

**DOI:** 10.3389/fpsyg.2025.1603872

**Published:** 2025-11-19

**Authors:** Chengrong Ling, Xuemei Li, Ling Zhao, Dongmei Zhao, Meng Qiu, Yingchun Li

**Affiliations:** 1 Department of General Surgery, The Second People’s Hospital of Yibin, Yibin, China; 2 Department of Emergency Medicine, West China Hospital, Sichuan University, Chengdu, China; 3 Department of Intensive Care Unit, The Second People’s Hospital of Yibin, Yibin, China; 4 Department of Nursing, The Second People’s Hospital of Yibin, Yibin, China

**Keywords:** female nurses, two children, work-family conflict, regulatory emotional self-efficacy, turnover intention

## Abstract

**Objective:**

This study examines the mediating role of regulatory emotional self-efficacy (RESE) in female nurses on the relationship between work-family conflict (WFC) and turnover intention (TI), focusing specifically on female nurses with two children in Tertiary Grade-A hospitals in Sichuan Province. By exploring how RESE influences the impact of WFC on nurses’ intentions to leave, the study aims to offer valuable insights for strategies to reduce nurse turnover and enhance the stability of the nursing workforce.

**Methods:**

A convenience sampling method was employed to recruit 1,370 female nurses with two children from 65 tertiary hospitals across 21 cities and prefectures in Sichuan Province. Participants completed a general information questionnaire along with the Work-Family Behavioral Role Conflict Scale (WFBRCS), the Regulatory Emotional Self-Efficacy Scale (RESES), and the Turnover Intention Scale (TIS) to assess WFC, RESE and TI.

**Results:**

Female nurses with two children reported a mean TIS score of 13.11 ± 3.93, with an average item score of 2.18 ± 0.66, reflecting a high level of TI. WFC was positively associated with TI (*r* = 0.485, *p* < 0.01), whereas RESE was negatively associated with TI (*r* = −0.382, *p* < 0.01). Furthermore, RESE was found to partially mediate the relationship between WFC and TI, suggesting that higher emotional self-efficacy can buffer the impact of WFC on nurses’ intentions to leave.

**Conclusion:**

It is recommended that hospital management implement a dual-track intervention system emphasizing both resource optimization and psychological empowerment. Practically, this could involve adopting flexible scheduling and increasing nursing staff to alleviate the strain of heavy workloads on nurses’ family responsibilities. Simultaneously, providing mindfulness training, regular psychological counseling, and emotional management workshops can strengthen nurses’ RESE, enabling them to better balance work and family demands, reduce TI, and promote the stability and sustainable development of the nursing workforce.

## 1 Introduction

As the population ages and global public health emergencies become increasingly frequent, the demand for nursing personnel continues to grow (Zhang et al., 2023), making nurse shortages a universal challenge for healthcare systems (Fasbender et al., 2019). In this context, reducing turnover rates is pivotal to stabilizing the nursing workforce. Global nurse turnover rates vary widely, ranging from 8 to 36.6% (Ren et al., 2024), while Chinese tertiary hospitals face an attrition rate of 13.1% (National Health Commission of the People’s Republic of China, 2023), which represents a high-risk threshold. Nurse turnover not only results in wasted training resources but also compromises the quality of patient care (Wu et al., 2014). Turnover intention (TI), as a key antecedent of actual turnover behavior (Hann et al., 2011), averages 28% globally (Xu et al., 2023) and reaches 49.58% among Chinese nurses (Wang et al., 2022), underscoring the urgent need for effective human resource management interventions.

China’s universal two-child policy, fully enacted in 2016, has substantially restructured China’s labor force demographic, disproportionately large impact on female employees, as cumulative empirical evidence shows each additional child is associated with a 10-percentage-point reduction in women’s labor force participation rates (Yang and He, 2022). This policy-driven trend creates acute organizational challenges for healthcare systems, particularly pronounced for nursing given that women constitute 98% of China’s nursing workforces (National Health Commission of the People’s Republic of China, 2023). Among these female nurses, 77% are of childbearing age (≤35 years) (National Health Commission of the People’s Republic of China, 2023), accounting for 40.79% of this group (Wu et al., 2022) have two children, and their turnover rate (63.73%) significantly exceeds that of the general nursing population (Li, 2018). Evidence indicates that the number of children is positively associated with turnover risk (Rasheed, 2019; Yao et al., 2024), particularly among nurses with two children (Huang et al., 2020), with work-family conflict arising from childcare explicitly identified as the top reason for female healthcare workers leaving their jobs (Lin and Li, 2025). Grounded in the conservation of resources (COR) theory (Hobfoll, 1989), these nurses face a resource loss spiral, including temporal conflicts between shift work and childcare, as well as emotional labor arising from both professional caregiving and family responsibilities. When losses (e.g., missed family events due to night shifts) outweigh gains (e.g., social support), TI emerges as a protective response to halt further depletion.

Work-family conflict (WFC) exemplifies this resource depletion. Under the two-child policy, a resource loss spiral emerges: night shifts drain temporal resources, while the dual demands of professional and family caregiving exhaust emotional resources, collectively exceeding nurses’ available resource thresholds; Emotional stress from balancing professional care and family responsibilities, compounded by disrupted career progression due to childbirth, contributes significantly to WFC. WFC refers to the tensions that arise when role demands in the work and family domains are incompatible (Beutell, 1985), encompassing both work-to-family interference (WIF) and family-to-work interference (FIW) (Netemeyer et al., 1996), and represents a critical manifestation of resource depletion. According to role conflict theory (Kahn et al., 1964), women face dual expectations from the workplace and family, intensifying WFCs, a phenomenon particularly pronounced in nursing. Empirical research indicates that the high-pressure, high-intensity nature of nursing work exacerbates WFC among nurses (Zhang et al., 2017; Grzywacz et al., 2006), which not only leads to emotional exhaustion but also serves as a significant predictor of TI (Hanoum et al., 2024), with WFC exerting a particularly strong negative impact (Zhou et al., 2020).

Regulatory emotional self-efficacy (RESE) may serve as a crucial internal resource that mitigates the impact of WFC on TI. RESE reflects an individual’s perceived ability to regulate and manage emotions (Bandura et al., 2003) and encompasses three key dimensions: These dimensions include perceived positive self-efficacy (POS), despondency/distress emotional self-efficacy (DES), and anger emotional self-efficacy (ANG) (Carletto et al., 2016). Managing despair from overtime and anger from patient conflicts align with COR’s tenet that individuals prioritize protecting vulnerable resources. Previous research indicates that WFC is negatively associated with RESE (Wang et al., 2023), whereas nurses with higher RESE tend to exhibit lower TI (Ling et al., 2024). These individuals are more likely to employ adaptive coping strategies, which help buffer stress and mitigate the adverse effects of WFC (Yaghoobi et al., 2019). However, most existing studies have focused primarily on external resources, such as workplace conditions (Jiang and Kira, 2024) and organizational support (Galanis et al., 2024), while giving insufficient attention to internal psychological resources like RESE. Additionally, international research has largely concentrated on nursing workforces in Western contexts, providing limited analysis of gender-role dynamics within the framework of China’s two-child policy.

**Figure 1:**
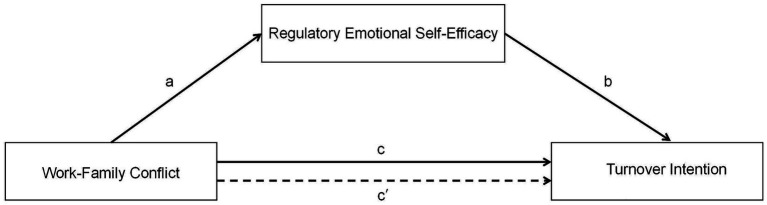
The theoretical model.

Building on COR theory, this study frames WFC as a resource threat spiral for female nurses with two children, adapted to China’s contexts. The dual-domain nature of WFC (WIF and FIW) directly reflects COR’s principle that stress occurs when key resources, such as time, energy, and emotional capacity, are depleted across competing domains. Under the influence of China’s traditional culture of “men do external things and women do internal things,” WFC’s influence on TI has been amplified. RESE is proposed as a resource caravan passageway that buffers this spiral, with its dimensions, DES, ANG, and perceived POS, aligning with COR’s idea that individuals prioritize protecting the resources most susceptible to loss. Notably, COR theory underemphasizes how China’s cultural role expectations worsen resource depletion, a factor rarely addressed in Western COR nursing studies. To fill this gap, our study integrates this element into COR: it links cultural norms to amplified WFC, notes scheduling constraints reduce resource protection, positions RESE as a buffer, and clarifies the interplay of WFC, RESE and TI in China’s context. Based on this, we present the conceptual model in Figure 1.

This study aims to examine the impact mechanisms of WFC and RESE on TI among female nurses with two children, while validating a culturally adapted COR framework. Theoretically, it contributes by refining COR’s application in non-Western healthcare settings. Practically, it provides a foundation for hospital administrators to design targeted psychological interventions and human resource management strategies. By mitigating TI associated with WFC in this nursing subgroup, the study supports nursing team stability and healthcare service quality improvement, while enhancing COR’s cultural sensitivity in the context of Chinese nursing research.

We propose the following hypotheses:

*H1*: WFC is positively associated with TI (path c).

*H2*: WFC is negatively correlated with RESE (path a).

*H3*: RESE is negatively associated with TI (path b).

*H4*: RESE mediates the effect of WFC on TI (path c’).

## 2 Materials and methods

### 2.1 Participants

This study employed a multi-center convenience sampling approach, recruiting 1,370 female nurses with two children from 65 tertiary hospitals across Sichuan Province between September and December 2023. Sichuan Province is one of the most populous provinces in China’s southwest region, administering 21 prefecture-level cities and autonomous prefectures. Tertiary hospitals are the core of China’s medical system, and the 65 tertiary hospitals involved in the study are distributed across all 21 prefecture-level administrative regions of Sichuan Province, ensuring the practical accessibility of sampling and the diversity of the sample. Inclusion criteria required participants to be registered female nurses employed by the hospital, with at least 1 year of clinical experience, two healthy children, and provision of informed consent. Exclusion criteria encompassed nurses undergoing standardized training programs, those with multiple pregnancies, divorced or widowed nurses, and individuals not currently engaged in clinical nursing or absent from such roles for over 6 months.

The sample size was determined through a rigorous, multi-step calculation to address both descriptive study requirements and the statistical demands of structural equation modeling (SEM). First, the sample size was calculated using the formula for descriptive studies, number of research variables multiplied by 5–10, totaling 26 variables: 18 demographic items, 2 dimensions from the WFC Scale, 3 from the RESE Scale (RESES), and 3 from the TI scale (TIS). Accordingly, the estimated sample size ranged from 130 to 260, which, after adjusting for a 20% attrition rate, increased to 312-468. Third, combine the sample size calculation requirements of structural equation model, to ensure the robustness of the model, the sample size should not be less than 200 cases (Wu, 2010). The study further expanded enrollment to ensure methodological rigor, the actual sample size reached 1,370 nurses, well above the minimum requirement. The study protocol received approval from the Medical Ethics Committee of The Second People’s Hospital of Yibin (Ethical Approval No.: 2023-225-01), and all participants provided written informed consent.

### 2.2 Research tools

#### 2.2.1 General information questionnaire

Drawing on a comprehensive literature review, the researchers developed a survey tailored to the study’s objectives. The questionnaire first ensured respondents met the core inclusion criterion of “being female nurses with two children” and then gathered detailed information on their demographic, professional, and family characteristics to explore potential influencing factors of core variables. The specific items included: hospital affiliation, age, professional title, educational background, department, position, personnel relationships, spouse’s occupation, years of work experience, monthly income, number of night shifts per month, the age and gender of both the first and second child, whether the second child was planned (designed to investigate the impact of fertility decision-making background and core study variables), the family’s primary caregiver, and total household income.

#### 2.2.2 Work-family behavior role conflict scale(WFBRCS)

This study utilized the Chinese version of the WFBRCS, translated by Sun et al. (2023) from the original 2019 version developed by Clark (2019). The scale includes two core subdimensions, 30 items in total, to measure bidirectional work-family conflict (WFC). The first is Work-to-Family Interference (WIF), which contains 15 items focusing on how work roles disrupt family life, with scores reflecting the frequency of work interfering with family responsibilities; the second is Family-to-Work Interference (FIW), which includes 15 items assessing how family roles hinder work performance, with scores capturing the frequency of family interfering with work duties. By assessing specific behavioral role conflicts, the scale offers a comprehensive understanding of the WFC process and informs the design of targeted interventions. All items are rated on a 5-point Likert scale (1 = “never,” 5 = “always”), with a theoretical mean score of 3 serving as the threshold—scores below 3 indicate low conflict, while scores of 3 or higher indicate high levels of WFC. In the present study, the scale demonstrated excellent reliability, with a Cronbach’s *α* coefficient of 0.952.

#### 2.2.3 Regulatory emotional self-efficacy scale (RESES)

The RESES, originally developed by Caprara et al. (2008) and later localized and revised by Sun et al. (2023), comprises three dimensions: perceived POS, DES, and ANG. The scale consists of 12 items, each rated on a 5-point scale, where 1 represents “very inconsistent” and 5 represents “very consistent,” with higher total scores reflecting greater RESE. Based on average item scores, RESE levels can be classified as low (≤2.5), medium (2.6–4.0), or high (≥4.1). In this study, the scale demonstrated excellent reliability, with a Cronbach’s *α* coefficient of 0.967.

#### 2.2.4 Turnover intention scale (TIS)

The TIS, originally developed by Michaels and Spector (1982) in 1982 and later translated and revised by Li and Li (2000), is designed to assess an individual’s level of TI. The scale consists of six items across three dimensions: TI I (Intention to Search for Alternative Jobs) includes 2 items measuring the willingness to actively seek other positions; TI II (Intention to Resign) contains 2 items assessing the specific intention to leave the current job; TI III (Evaluation of Job Mobility) includes 2 items reflecting the attitude toward job switching. All items are rated on a 4-point Likert scale (1 = never, 2 = rarely, 3 = occasionally, 4 = frequently), yielding a total score range of 6 to 24. Higher scores indicate stronger TI. Based on average item scores, TI is categorized as very low (≤1), low (>1 to ≤2), high (>2 to ≤3), and very high (>3). In the present study, the scale demonstrated good reliability, with a Cronbach’s *α* coefficient of 0.838.

### 2.3 Data collection methods

Data were collected using the Questionnaire Star electronic platform. Prior to the formal survey, a pilot study was conducted in August 2023 to refine questionnaire wording and structure based on feedback. The research team held online meetings with nursing departments from all 65 participating hospitals to explain the study objectives (Ethics Approval No. 2023-225-01), anonymity safeguards, and data usage. After obtaining institutional authorization, standardized training was provided to nursing liaison officers at each hospital, including demonstrations of questionnaire completion, answers to frequently asked questions, and guidance on optimal administration timing, such as avoiding shift-change periods. During this training, we explicitly required liaison officers to only distribute the questionnaire link to nurses confirmed to meet the “having two children” criterion and to refuse access to ineligible nurses, ensuring the initial participant pool targeted the core study population. The platform incorporated quality control measures, including mandatory responses and restriction to one submission per IP address. All nurses voluntarily completed the questionnaires anonymously after providing informed consent. During the data collection period (September–December 2023), two researchers monitored submissions in real-time and tracked response rates for each hospital. A total of 1,392 questionnaires were collected, and to further ensure eligibility, we conducted post-collection screening: cross-checking with mandatory “age of the first child” and “age of the second child” items in the general information questionnaire (excluding those with blank, contradictory, or single-child-implying responses); ultimately, 22 invalid responses were excluded (9 for not meeting the “having two children” criterion, 13 for patterned responses or logical inconsistencies), resulting in 1,370 valid questionnaires and a 98.4% validity rate.

### 2.4 Statistical methods

After double-checking the data, questionnaire results were imported into SPSS 27.0 for statistical analysis, with *p* < 0.05 considered statistically significant. The normality of variables was assessed using Q-Q plots, and scale scores along with other quantitative data were reported as mean ± standard deviation. Descriptive statistics, including frequencies and percentages, were used to summarize demographic information. Pearson correlation coefficients were calculated to examine relationships among WFC, RESE, and TI. A structural equation model (SEM) was constructed in AMOS 24.0 to investigate the mediating role of RESE in the relationship between WFC and TI among female nurses with two children, and the significance of the mediation effect was tested using the Bootstrap method.

## 3 Results

### 3.1 General information about the subjects

The participants’ general information are presented in Table 1.

**Table 1:** General data of female nurses with two children (*n* = 1,370).

Project	Group	Number (*n*)	Percentage (%)
Age	<30	85	6.2
	31–35	627	45.8
	36–40	486	35.5
	41–45	150	10.9
	>45	22	1.6
Professional title	Junior or below	383	28.0
	Intermediate	887	64.7
	Senior and above	100	7.3
Education	College or below	108	7.9
	Bachelor’s degree	1,248	91.1
	Master’s or above	14	1.0
Years of work experience	Years <5	22	1.6
	5 ≤ years<10	186	13.6
	10 ≤ years<15	703	51.3
	15 ≤ years<20	314	22.9
	Years ≥20	145	10.6
Employment status	Permanent staff	274	20.0
	Agency personnel	20	1.5
	Contract-based	1,076	78.5
Department	Internal Medicine	457	33.4
	Surgery	416	30.4
	Obstetrics/Gynecology	78	5.7
	Pediatrics	65	4.7
	Others	354	25.8
Position	Staff nurse	1,160	84.7
	Head nurse	141	10.3
	Nursing department staff	24	1.8
	Others	45	3.3
Night shifts per month	None	465	33.9
	1–5	528	38.5
	6–10	307	22.4
	>10	70	5.1
Spouse’s occupation	Unemployed/Homemaker	40	2.9
	Enterprise/Institution employee	382	27.9
	Military personnel	26	1.9
	Self-employed	161	11.8
	Government/Party/Enterprise leader	105	7.7
	Others	656	47.9
Average monthly income (Yuan)	<3,500	42	3.1
	3,501–5,500	352	25.7
	5,501–10,000	865	63.1
	>10,000	111	8.1
Gender of the first child	Male	655	47.8
	Female	715	52.2
Age of the first child	<3	16	1.2
	3-<6	186	13.6
	6-<10	576	42.0
	≥10	592	43.2
Gender of the second child	Male	704	51.4
	Female	666	48.6
Age of the second child	<3	389	28.4
	3-<6	554	40.4
	6-<10	389	28.4
	≥10	38	2.8
Whether the second child was planned	Yes	881	64.3
	No	489	35.7
Primary caregiver for the children	Oneself	126	9.2
	Oneself and spouse	338	24.7
	Hired nanny	27	2.0
	Paternal grandparents	515	37.6
	Maternal grandparents	325	23.7
	Others	39	2.8
Household monthly income (Yuan)	<5,000	30	2.2
	5,001–10,000	431	31.5
	10,001–15,000	486	35.5
	15,001–20,000	268	19.6
	>20,000	155	11.3

### 3.2 Common method bias

To evaluate potential common method bias, Harman’s single-factor test was conducted. Factor analysis using SPSS identified eight factors with eigenvalues greater than 1, which together accounted for 68.02% of the total variance. The first factor explained 31.00% of the variance, below the critical 40% threshold, indicating that no significant common method bias was present in the questionnaire data.

### 3.3 Female nurses with two children WFBRC, RESE, and TI scores

The total scores and dimension scores of WFBRC, RESE, and TI are shown in Table 2

**Table 2:** WFBRC, RESE, and TI scores (*n* = 1,370).

Project	Number of items (items)	Theoretical maximum score	Score (score, mean ± SD)	Average item score (Score, mean ± SD)
WFBRC	30	150	83.01 ± 19.29	2.77 ± 0.64
WIF	15	75	50.63 ± 10.81	3.38 ± 0.72
FIW	15	75	32.38 ± 11.17	2.16 ± 0.74
RESE	12	60	44.18 ± 7.79	3.68 ± 0.65
POS dimension	4	20	16.63 ± 2.83	4.16 ± 0.71
DES dimension	4	20	14.05 ± 3.15	3.51 ± 0.79
ANG Dimension	4	20	13.5 ± 3.42	3.37 ± 0.85
TI	6	24	13.11 ± 3.93	2.18 ± 0.66
TI I dimension	2	8	3.88 ± 1.71	1.94 ± 0.86
TI II dimension	2	8	4.67 ± 1.41	2.33 ± 0.71
TI III dimension	2	8	4.56 ± 1.29	2.28 ± 0.65

### 3.4 Correlation among WFC, RESE, and TI in female nurses with two children (aligned with H1, H2, H3)

The study found that WFC among female nurses with two children was positively correlated with TI (*r* = 0.485, *p* < 0.01), while RESE was negatively correlated with TI (*r* = −0.382, *p* < 0.01). Additionally, WFC was negatively correlated with RESE (*r* = −0.418, *p* < 0.01). Detailed correlation coefficients are presented in Table 3. The results of the study verified the validity of hypotheses 1–3.

**Table 3:** Correlation analysis of WFC, RESE and TI of female nurses with two children (*n* = 1,370, r value).

Variables	WIF	FIW	WFBRCS score	POS	DES	ANG	RESES score	TIS score
WIF	1	–	–	–	–	–	–	–
FIW	0.540**	1	–	–	–	–	–	–
WFBRCS	0.873**	0.882**	1	–	–	–	–	–
POS	−0.148**	−0.195**	−0.196**	1	–	–	–	–
DES	−0.346**	−0.294**	−0.364**	0.249**	1	–	–	–
ANG	−0.343**	−0.294**	−0.362**	0.263**	0.482**	1	–	–
RESES	−0.381**	−0.352**	−0.418**	0.641**	0.782**	0.807**	1	–
TIS	0.412**	0.440**	0.485**	−0.239**	−0.311**	−0.301**	−0.382**	1

*p < 0.01, **p < 0.01.

### 3.5 The mediating effect of RESE of female nurses with two children on WFC and TI (aligned with H4)

In this study, WFC served as the independent variable, TI as the dependent variable, and RESE as the mediating variable. Stepwise regression analysis in SPSS indicated that RESE partially mediates the relationship between WFC and TI, with the explained variance increasing from 23.6 to 27.4% (*p* < 0.001). Detailed results are presented in Table 4.

**Table 4:** Analysis of the mediating effect of RESE between WFC and TI.

Step	Independent variable	Dependent variable	Standardized equation	R2	B	Standard error	t	*p*
1	WFC (X)	TI (Y)	Y = 0.485*X	0.236	0.099	0.005	20.538	<0.001
2	WFC (X)	RESE (M)	M = −0.418X	0.174	−0.152	0.009	−17.003	<0.001
3	WFC (X)	TI (Y)	Y = 0.395*X-0.217*M	0.274	0.080	0.005	15.577	<0.001
	RESE (M)				−0.121	0.014	−8.544	<0.001

**Figure 2:**
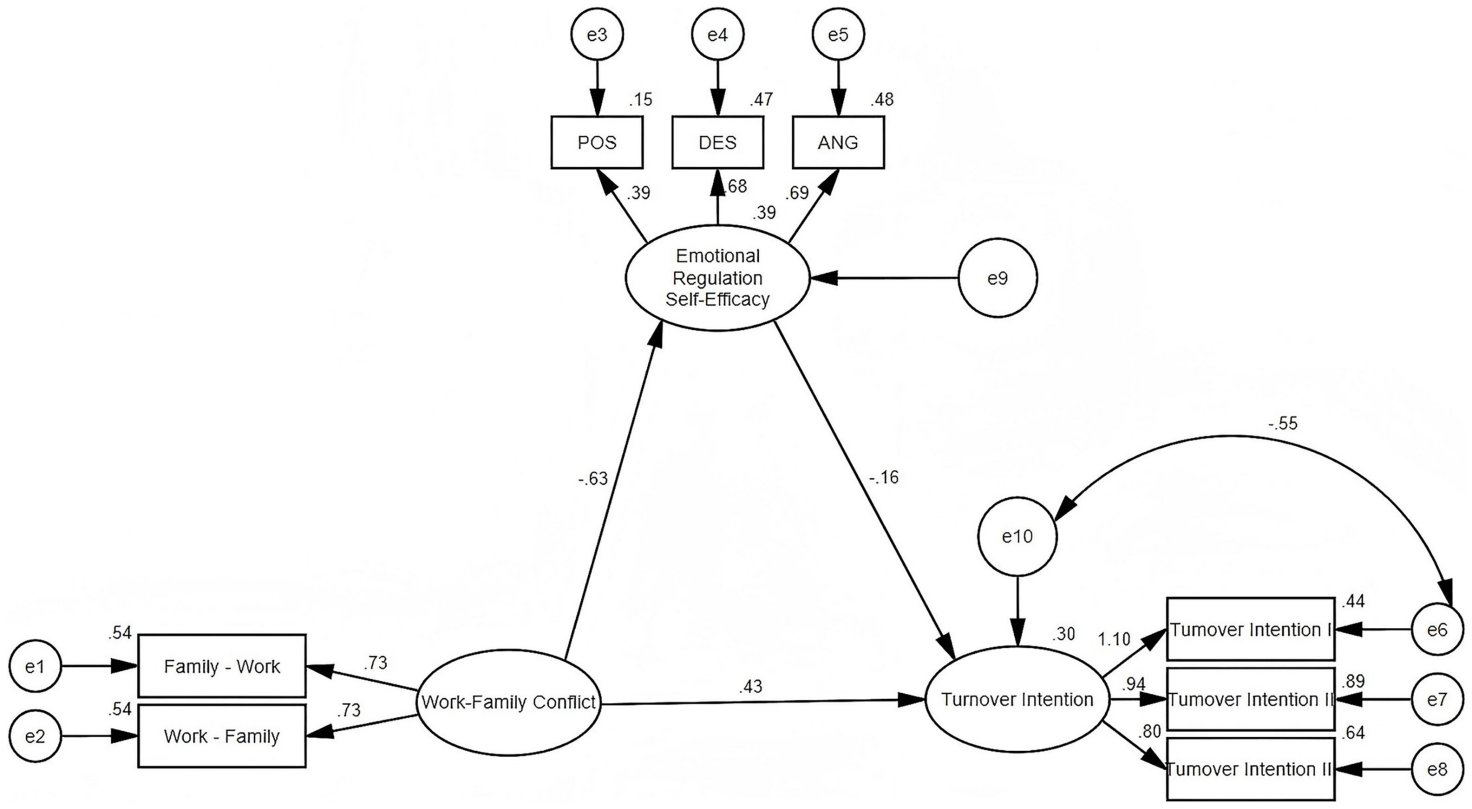
Model diagram of the mediation effect of RESE between WFC and TI in female nurses with two children.

Based on the aforementioned findings, we employed AMOS 24.0 to construct a structural equation model (SEM) following a two-stage analytical procedure. First, confirmatory factor analysis (CFA) was performed to validate the measurement model, demonstrating adequate psychometric properties with factor loadings ranging from 0.579 to 0.92 (all *p* < 0.01), satisfactory composite reliability (CR > 0.7), and acceptable convergent validity (AVE > 0.5) (Bagozzi et al., 1981). Discriminant validity was confirmed as the square roots of AVEs for all latent variables exceeded their inter-construct correlations in accordance with the Fornell-Larcker criterion. Subsequently, maximum likelihood estimation was applied to test and refine the hypothesized SEM, yielding final model fit indices that met recommended standards (Kang and Ahn, 2021) (see Table 5) with the structural relationships illustrated in Figure 2.

**Table 5:** Standards and results for fitting indices in SEM.

Goodness -of-fit index	Index value indicators	Criterion	Fit degree fitting
χ^2^/df	3.296	<5.000	good
CFI	0.990	>0.900	good
AGFI	0.978	>0.900	good
GFI	0.990	>0.900	good
NFI	0.986	>0.900	good
RMSEA	0.041	<0.080	good
TLI	0.983	>0.900	good

The Bootstrap method was used to examine the mediating effect of RESE on the relationship between WFC and TI. Bootstrap sampling was conducted with 5,000 iterations and a 95% confidence interval, with mediation considered significant if the interval did not include zero. The 95% confidence interval for the mediating effect was 0.050–0.157, excluding zero, indicating that RESE partially mediates the relationship between WFC and TI among female nurses with two children, accounting for 19.48% of the mediating effect (Table 6). This means that the validity of the H4 hypothesis is verified.

**Table 6:** Analysis of the mediation effect of RESE between WFC and TI.

Type of effect	Path	St. Estimate	SE	95%CI	*p*	Effect ratio%
Direct effect	WFC → TI	0.426	0.046	0.340–0.522	0.002	80.52%
Indirect effect	WFC → RESE → TI	0.103	0.027	0.050–0.157	0.002	19.48%
Overall benefits	WFC → TI	0.529	0.030	0.468–0.586	0.002	100.00%

## 4 Discussion

### 4.1 Current status of WFC, RESE, and TI among female nurses with two children

#### 4.1.1 Status of WFC

Our study found that the level of WFC among female nurses with two children was higher than that of Gan et al. regarding the general nurse population in China (Gan et al., 2025), consistent with COR theory: rotating night shifts compete directly with childcare needs, such as disrupted sleep versus school routines, while emotional labor is divided between patient care and parenting responsibilities (Hwang and Yu, 2021). Recent research indicates that nurses endure dual sources of emotional exhaustion arising from professional empathy at work (Huaman et al., 2023). The data reveal a significant imbalance between WIF (50.63 ± 10.81) and FIW (32.38 ± 11.17, *p* < 0.001). This discrepancy stems from the unique nature of nursing work: Female nurses with two children face rigid work requirements such as fixed hospital schedules and unexpected overtime (Chen et al., 2022). These demands make it difficult to flexibly allocate working hours and energy, thereby exerting greater pressure on family care responsibilities. Moreover, in China’s public hospitals, where nurses face expanding responsibilities including clinical care, teaching, and research (Cooper et al., 2019), these tasks take up a lot of downtime. In contrast, FIW had a weaker impact, which is associated with the “work-first” professional culture in China’s healthcare settings (Pajakoski et al., 2025). Most nurses proactively adjust family matters to minimize interference with work. Although this approach may temporarily enhance productivity, chronic role overload elevates turnover risk by straining family relationships (Gehri et al., 2023).

#### 4.1.2 Current situation of RESE

This study found that the score of RESE among female nurses with two children exhibited above-average (Sun et al., 2023), this result is consistent with the findings of Wang et al., who conducted a study on infectious disease nurses in China (Wang et al., 2023), suggesting an adaptive capacity developed through prolonged dual-role exposure. This prolonged dual training may passively enhance their emotional regulation capabilities, which aligns with Bandura’s “practical reinforcement efficacy” theory (Bandura, 1977). However, their lowest scores were observed in anger management ANG, the possible reasons for this result are: First, in professional settings, nurses continuously cope with emotional events such as patient anxiety and conflicts in family communication (Pace et al., 2023). Long-term passive exposure to negative emotions easily depletes their emotional resources for anger regulation. Second, the complex task of childcare has left nurses extremely exhausted, physical fatigue further impairs their ability to perceive and manage anger (Getie et al., 2025). From the perspective of COR theory, these two factors essentially reflect the continuous depletion of emotional and physical resources among this group. Thus, it is suggested that healthcare institutions prioritize targeted interventions, such as providing regular anger regulation training to replenish emotional resources.

#### 4.1.3 Current situation of TI

This study revealed elevated TI among female nurses with two children, with a mean item score of 2.18 ± 0.66. This score exceeds both the established benchmarks (Clark, 2019) and the TI score of nurses in a Chinese hospital reported by Zhang et al. (2023). Several factors likely contribute to this finding. First, 64.7% of participants held mid-level professional titles, reflecting high professional competence and career maturity; according to human capital theory (Yakusheva et al., 2024), such individuals may perceive resignation as a strategic career decision rather than a passive response to dissatisfaction. Second, 38.5% of nurses worked night shifts at least five times per month, interfering with family responsibilities (He et al., 2024). Within the context of traditional Chinese gender norms, where men are expected to work outside the home and women manage domestic duties, female nurses may be more inclined to leave their jobs to prioritize family obligations. Third, limited career advancement opportunities elevate turnover risk (Şahin et al., 2025). Specifically, among the three dimensions of TI, the subdimension “motivation for seeking other jobs” (TI II) had the highest average score (2.33 ± 0.71). This pattern is likely driven by the fact that 92.7% of participants held mid-level professional titles, which creates a bottleneck for further career progression. Compounding this, 78.8% of the nurses had a second child under 6 years old, a stage marked by particularly intensive parenting demands that may further amplify their inclination to seek other childcare support jobs. These findings suggest that nursing managers should closely address the unique needs of female nurses with two children and implement proactive measures to enhance job satisfaction and retention.

### 4.2 Correlation among WFC, RESE, and TI in female nurses with two children

#### 4.2.1 The relationship between WFC and TI

Grounded in COR theory, this study demonstrates that WFC significantly depletes the personal and emotional resources of female nurses with two children, ultimately elevating their TI (*β* = 0.42, *p* < 0.01), consistent with existing evidence on nursing stress and attrition (He et al., 2024). Nursing, inherently demanding due to high work intensity and disruptive shift schedules (Yao et al., 2024), conflicts with the substantial resource demands of raising two children, including time, energy, and emotional labor. When these competing strains exceed nurses’ resource thresholds, COR theory’s “resource loss spiral” is triggered: the chronic depletion of critical resources, such as work-life balance and psychological stamina, diminishes job satisfaction and amplifies withdrawal behaviors like TI, as nurses seek to halt further resource erosion. Notably, the asymmetrical impact of FIW (*r* = 0.440) versus WIF (*r* = 0.412) reflects the defensive prioritization of family resources within Chinese cultural contexts. According to COR theory, individuals safeguard non-replenishable resources, such as maternal roles and family harmony, when threatened; consequently, unavoidable family crises, such as a child’s illness, often lead nurses to compromise career resources, including job retention, to preserve familial stability rather than risk irreversible damage to valued kinship roles (Yildiz et al., 2021). The administrator can develop a flexible shift system for the group of nurses, such as avoiding spouse shift overlaps, requesting daytime shifts during children’s exams. This measure does not require additional technical input, and the current intelligent shift system can complete it, which directly reducing turnover due to work-family conflict.

#### 4.2.2 The relationship between RESE and TI

This study demonstrates that RESE negatively predicts TI, consistent with prior research in the Chinese context showing that emotional regulation self-efficacy (RESE) significantly influences nurse burnout (Moloney et al., 2018). From a COR theory perspective, RESE functions as a key “personal resource caravan,” a modifiable internal resource that buffers against resource loss spirals triggered by WFC. Dimensional analysis further highlights the DES subscale as the most influential protective factor, suggesting that nurses adept at managing distress, for example, through cognitive reappraisal, are less likely to resign despite the dual-role pressures of parenting and shift work. Self-determination theory (Song et al., 2023) supports this connection, positing that chronic unregulated emotions foster negative cognitions that elevate turnover risk. This aligns with COR’s proposition that individuals prioritize protecting resources most susceptible to depletion; for female nurses with two childrens, distress from unmet family demands or work overload is a primary vulnerable resource, which makes DES a critical buffer. Accordingly, interventions such as mindfulness training and emotion-focused workshops (Karahan Kaplan et al., 2024) may enhance RESE, particularly DES skills, helping nurses navigate the tensions between childcare and professional responsibilities while reducing attrition.

#### 4.2.3 The relationship between WFC and RESE

This study confirms a negative correlation between WFC and RESE, demonstrating that persistent conflicts erode nurses’ confidence in managing emotions, a pattern consistent with Wang et al. (2023) and French studies on stress-induced self-efficacy reduction (Murtaza et al., 2021). From a COR theory lens, chronic WFC acts as a “resource drain” that weakens RESE: scenarios like guilt from missing a child’s parent-teacher conference or anxiety over declining work performance due to family demands deplete emotional resources, in turn reducing nurses’ perceived ability to manage future emotional challenges, as supported by Australian interventions showing that resilience training can restore coping capacity (Foster et al., 2024). By concurrently mitigating external stressors and strengthening internal resources, healthcare systems can disrupt the WFC-RESE depletion cycle, transforming it into a positive reinforcement loop whereby improved conflict management enhances emotional competence and reduces vulnerability to future stress.

### 4.3 The mediating effect of RESE of female nurses with two children on WFC and TI

The mediation analysis indicates that RESE partially mediates the relationship between WFC and TI, consistent with COR theory, which posits that stress arises when individuals’ critical resources are threatened or depleted (Hobfoll, 1989). In this context, WFC not only directly predicts TI but also indirectly exacerbates it by reducing RESE, a key psychological resource that buffers against emotional exhaustion. For nurses managing dual responsibilities, such as childcare and shift work, prolonged WFC depletes finite resources, including time and emotional energy, and as RESE declines, their ability to manage the resulting distress weakens, further amplifying TI as a protective response to halt resource loss.

Research shows that when balancing family responsibilities and professional commitments, individuals inevitably experience negative emotions such as sadness, frustration, anxiety, anger, and irritability (Feng et al., 2024; Guo et al., 2025). Importantly, RESE moderates this cycle: individuals with high RESE demonstrate greater competence in cognitive reappraisal and negative emotion regulation (Meng et al., 2024; Spitzen et al., 2022), particularly in managing depression (DES, *λ* = 0.68) and anger (ANG, λ = 0.69), which facilitates faster recovery from distress (Yu et al., 2024). SEM further highlights this asymmetry, showing that the regulation of negative emotions exerts an influence 1.7 times stronger than that of positive emotion expression (POS, λ = 0.39). From a COR theory perspective, this asymmetry reflects the fact that WFC primarily induces negative affect, making DES and ANG the most relevant resources for mitigating resource loss. This finding aligns with prior evidence that the consequences of WFC are driven by unmanaged negative affect (French and Allen, 2020), and it extends COR theory by identifying specific RESE dimensions that act as targeted buffers for this policy-specific subgroup.

Notably, while RESE attenuates the WFC → TI pathway (*β* = 0.426), its partial mediation underscores the need for concurrent structural and psychological interventions, which is consistent with COR’s “resource protection + resource building” framework (Hobfoll, 1989). Structural measures (flexible scheduling, on-site childcare support) reduce WFC at its source and curb initial resource depletion; targeted RESE training (integrated into in-service education) builds internal resources to buffer residual WFC. To complement these policies, institutional reforms should integrate targeted RESE-training programs into in-service education. For instance, they can organize emotional regulation training workshops twice a month, which are led by clinical psychologists. These seminars will cover modules including mindfulness practice, cognitive-behavioral techniques for anger management during high-stress shifts, emotional decompression after balancing dual work-family demands, and positive self-talk to alleviate turnover anxiety. This approach leverages existing hospital conference rooms, directly reinforces RESE’s mediating role in offsetting WFC-induced resource depletion, and aligns with national policies on nurse support and professional development. By extending COR theory to gendered occupational stress, this study identifies RESE as a trainable resilience factor. Healthcare systems seeking to reduce attrition must therefore address both systemic drivers of WFC and individual psychological resources, promoting sustainable nursing careers and higher standards of patient care.

## 5 Limitations

This study has several limitations. First, the sample comprised female nurses with two children from tertiary hospitals in Sichuan Province, China; due to regional and cultural factors, the findings may have limited generalizability internationally. Future studies should include more diverse regions and hospital levels to enhance representativeness. Second, although the cross-sectional design allowed for statistical validation of relationships among variables, it cannot establish causality. Future research employing longitudinal tracking or experimental designs is recommended to clarify temporal sequences and strengthen causal inferences in this field. Third, this study did not examine the influence of spousal support, such as participation in co-parenting, on resource compensation. Future research should incorporate marital interaction variables to better understand how family dynamics contribute to nurses’ WFC, RESE, and TI. Fourth, our study did not statistically control for potential confounders such as age, work experience, workplace type, marital status, professional titles, or night shift frequency. For example, nurses with longer tenure may have developed better coping strategies for WFC, potentially attenuating the observed effect sizes. Similarly, unmeasured differences in workplace support could bias the relationship between self-efficacy and turnover intention. Future studies should incorporate these variables into hierarchical regression or multi-group SEM analyses to validate our findings under more rigorous conditions.

## 6 Conclusion

This study found that female nurses with two children working in public hospitals in Sichuan Province exhibit elevated TI, with WFC positively correlated and RESE negatively correlated with TI. Moreover, WFC was negatively associated with RESE, which partially mediated its effect on TI. In response, hospital management should establish a dual-track intervention system emphasizing both resource optimization and psychological empowerment. Flexible scheduling and increased staffing can mitigate the negative impact of excessive workloads on nurses’ family lives, while mindfulness training, regular psychological counseling, and emotion management workshops can enhance nurses’ emotional regulation skills, enabling them to better navigate WFC, reduce TI, and support workforce stability and sustainable development. This study advances COR theory by empirically demonstrating that RESE functions as a modifiable energy resource that mitigates the resource loss spiral among female nurses with two children. Academically, this fills two gaps: prior COR studies in nursing focused on fixed personal resources (not modifiable emotional resources like RESE) and overlooked the “female nurses with two children” subgroup, while theoretically, it expands COR’s “resource types” (including modifiable state resources), refines the bidirectional “resource loss spiral” mechanism for dual-role nurses, and enhances the theory’s cultural adaptability to Chinese contexts; practically, it guides nursing management, policy formulation, and nursing education, addressing current gaps in each domain.

## Data Availability

The raw data supporting the conclusions of this article will be made available by the authors, without undue reservation.
